# International Clinics

**Published:** 1891-08

**Authors:** 


International Clinics. A Quarterly of Clinical Lectures on Medicine, Surgery, Gynecology, Pediatrics, Neurology, Dermatology, Laryngology, Ophthalmology, and Otology. By Professors and Lecturers in the leading medical colleges of the United States, Great Britain, and Canada. Edited by John M. Keating, M. D., Philadelphia; J. P. Crozer Griffith, M. D., Philadelphia; J. Mitchell Bruce, M. D., F. R. C. P., London, Eng., and David W. Finlay, M. D., F. R. C. P., London, Eng., April 18, 1891. Large octavo; pp. viii.—350. Illustrated. Philadelphia: J. B. Lippincott Company. Price, cloth, $2.75 ; half leather, $3.00.

The object of this periodical, as stated in the preface, is to furnish a complete post-graduate course of medical instruction. The object is a very commendable one, but whether it will bear out the hopes of its editors remains to be seen. Judging from the variety of subjects dwelt upon, and the high rank of the writers, it promises good things. Among the articles treated in the first volume may be mentioned : Acromegaly, by Dr. Ross ; Polyemia or Plethora in its Relation to Inflammation, by William Henry Porter, M. D.; Sore Throat, by Christopher Heath, F. R. C. S.; The Treatment of Cough in Phthisis, by J. Mitchell Bruce ; Modern Methods in Surgical Operations, by W. W. Keen ; Ulcers, by D. W. Cheever ; Scirrhus of the Breast, by Chas. T. Parkes ; the Early Diagnosis of Pregnancy, by M. D. Mann ; Tonsillar Diphtheria, by F. Forchheimer ; Different Types of Paralysis in Young Children, by L. C. Gray ; Functional Nervous Troubles, by B. Sachs ; Neurotomia and Athetoid Spasm, by C. K. Mills ; Alcoholic Paralysis, by David Ferrier, etc., etc. The plates and figures are excellent, and aid materially in the usefulness of the book ; the typography is faultless.

